# Acrylic tooth splint—An immediate provisionalisation following tooth extraction; a series of case reports

**DOI:** 10.1002/ccr3.7583

**Published:** 2023-06-20

**Authors:** Fara Azwin Adam, Farah Syazwani Mohamd Tarmizi, Chui Ling Goo

**Affiliations:** ^1^ Center for Periodontology Studies, Faculty of Dentistry Universiti Teknologi MARA, Jalan Hospital Sungai Buloh Malaysia; ^2^ Oral Health Division Ministry of Health Malaysia Putrajaya Malaysia; ^3^ Unit Prosthodontics, Department of Restorative Dentistry Faculty of Dentistry, Universiti Kebangsaan Malaysia, Jalan Raja Muda Abdul Aziz Kuala Lumpur Malaysia

**Keywords:** acrylic tooth, cost‐effective, extracted tooth, immediate tooth replacement, temporisation, tooth‐splint

## Abstract

**Key Clinical Message:**

Numerous techniques for provisionally replacing a single tooth at the aesthetic zone while planning for future dental implant placement, while soft and hard tissues heal, are critical in restorative treatment strategy. The available materials, simplicity, cost, and impact on the potential implant location should be considered when evaluating the treatment choices.

**Abstract:**

Replacement of a single anterior tooth in the aesthetic zone while planning for future implant placement is crucial to the restorative treatment plan. Several methods exist for immediate provisionalisation of the extracted tooth while waiting for soft and hard tissue healing. Although there's a myriad of possible provisionalisation methods available, each option has its advantages and disadvantages. The treatment options should weigh various factors such as the available materials, ease of fabrication, costs, and the effect on the future implant site. This article describes three clinical cases demonstrating a simple yet cost‐effective technique to temporarily replace an extracted single anterior tooth, enhancing patient satisfaction and increasing compliance before receiving the definitive implant restoration. The pro and cons for each treatment option available as opposed to the technique involved in the three cases used are also described.

## 1 INTRODUCTION

When a tooth in the aesthetic zone often requires an extraction for various reasons, the patient's primary concern is, “Will I go home with a tooth replacement?” While immediate implants are possible in specific clinical situations, this method may not be suitable for periodontally involved teeth with substantial bone loss before extraction (Levine et al., 2017). Thus, in such situations, bone grafting procedures may be indicated during the extraction stage itself to prepare for a suitable implant site before implant placement.^1^ After the extraction and alveolar ridge preservation procedures, an interim prosthesis would be necessary.^2^ There are many treatment options to consider for the immediate replacement of the extracted tooth.^3^, ^4^, ^5^, ^6^ One such option utilizes the patient's own extracted tooth, which is decoronated and used as a “pontic” secured with a high molecular weight polyethylene fiber^7^, ^8^ or a wire^9^ to adjacent teeth. Other options include using an acrylic denture tooth held with a fiber‐reinforced splint,^10^ anterior fixed provisional restoration with composite resin‐reinforced with a leno‐woven polyethylene ribbon^11^, ^12^ as well as immediate dentures which is the most common method.^13^, ^14^ This case series will discuss the clinical and laboratory steps of a simple and inexpensive replacement of a soon‐to‐be extracted periodontally compromised anterior tooth.

### 1.1 Case 1

**FIGURE 1:**
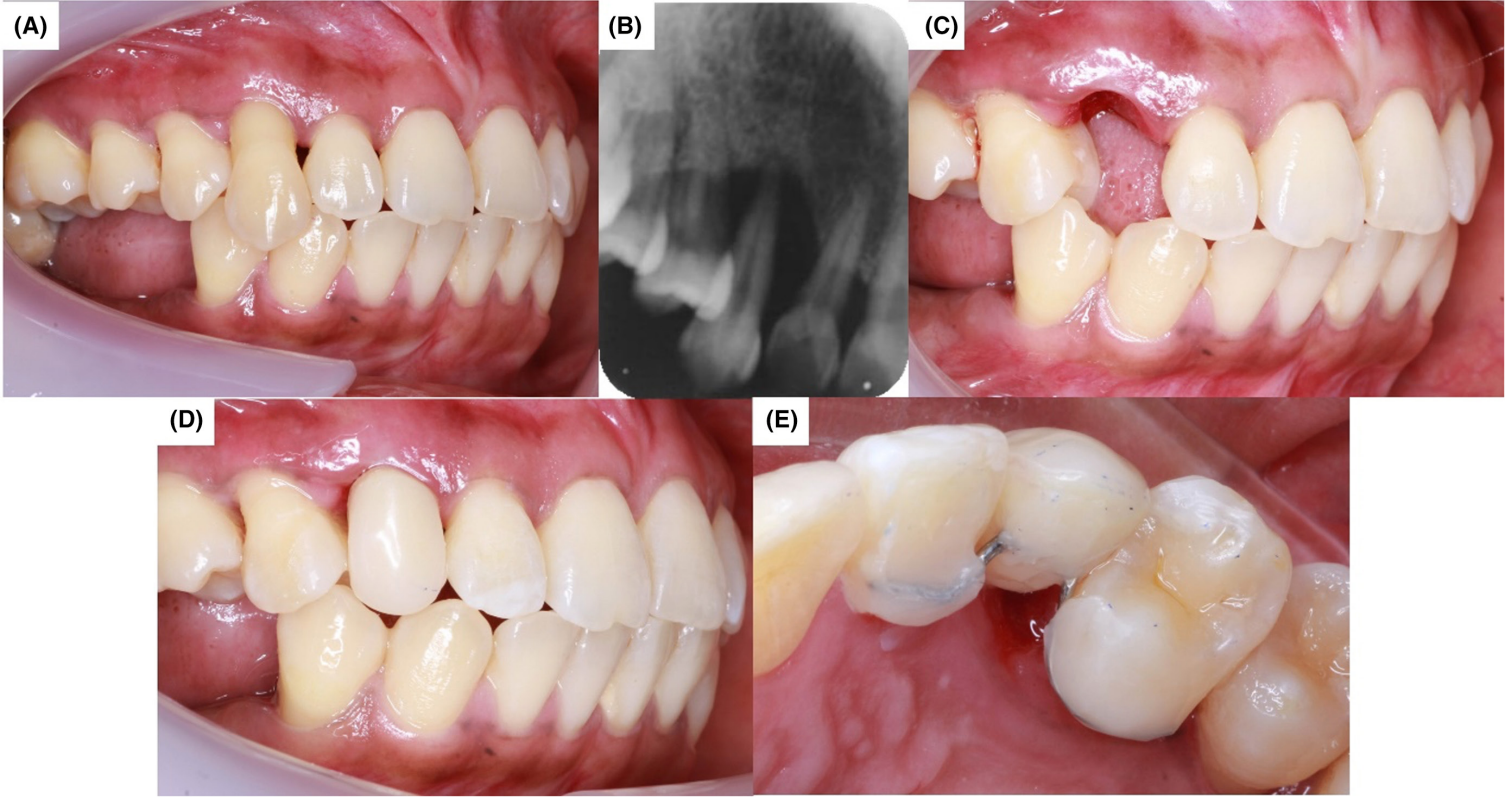
Clinical photographs from case 1. (A) Right lateral view showing an over‐erupted, periodontally involved tooth 13. (B) Periapical radiograph of tooth 13 during the pre‐operative stage. (C) Right lateral view following atraumatic extraction. (D) Right lateral view showing acrylic tooth splint, with an acceptable size, shade, and not in occlusion. (E) Occlusal view showing cemented acrylic tooth splint to adjacent teeth.

A 42‐year‐old female patient complained of a mobile and sensitive upper right canine tooth (Figure 1A). There is no contributing medical and social history. Dental history revealed an episode of trauma in the maxillary anterior region 5 years ago. Upon clinical examination, the right maxillary canine showed grade III mobility, with periodontal pocketing ranging from 6 to 8 mm and gingival recession of 3–6 mm on six points pocket charting. Pulp sensibility tests were conducted with no response from that right maxillary canine. The periapical radiograph revealed a total loss of bony support for that tooth (Figure 1). The tooth was diagnosed with localized apical periodontitis. The prognosis was poor due to the loss of bone support up to the root length. The only treatment option was extraction, which was done atraumatically, as shown in Figure 1C and replaced with a bonded acrylic tooth splint (Figure 1D,E).

### 1.2 Case 2

**FIGURE 2:**
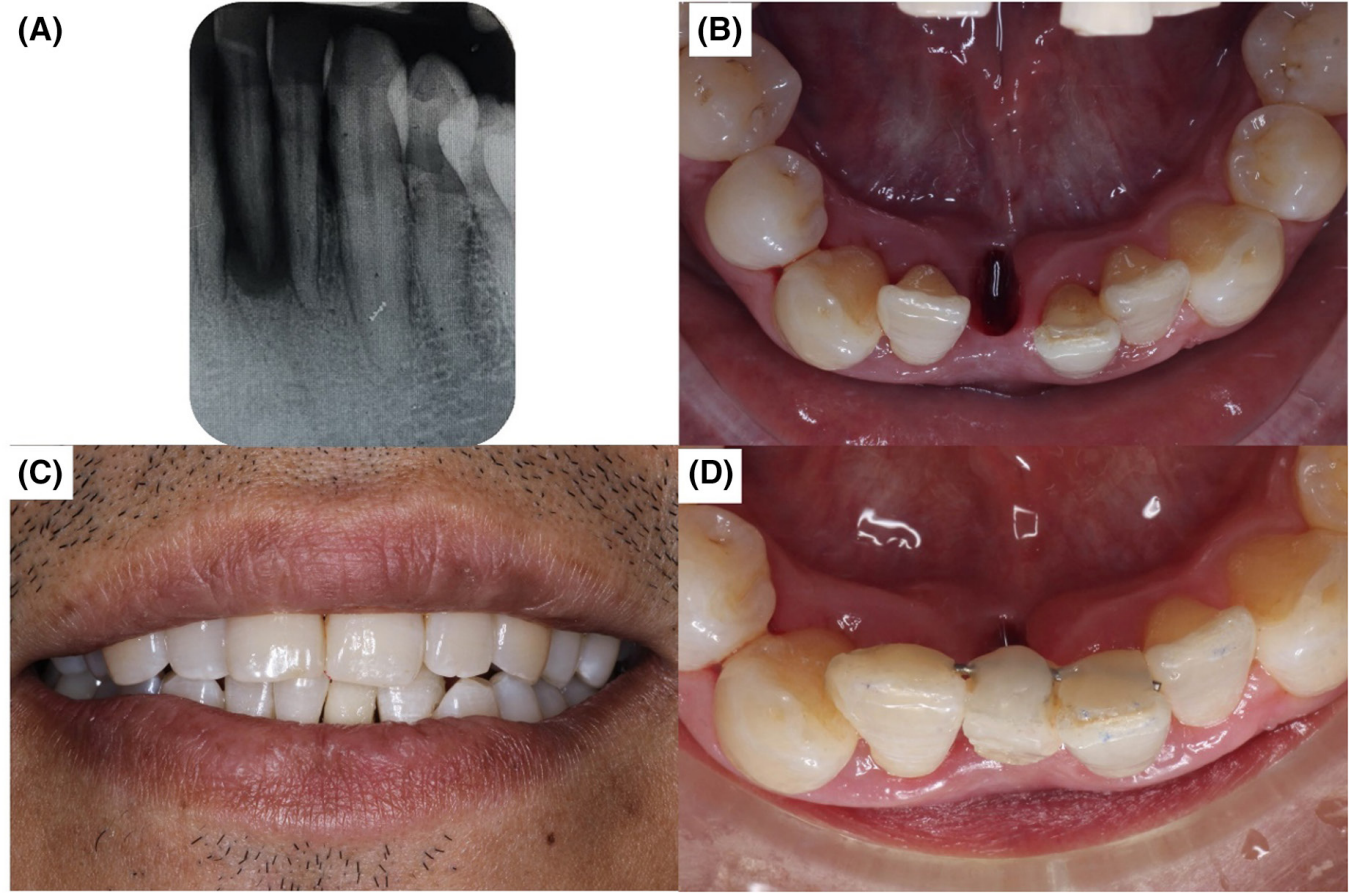
Clinical photographs from case 2. (A) Periapical radiograph of tooth 41 taken during the pre‐operative stage. (B) Occlusal view‐showing socket of tooth 41 following atraumatic extraction. (C) Frontal view showing bonding of the acrylic tooth splint on 41 with acceptable aesthetics. (D) Occlusal view after bonding acrylic tooth splint on 41 to accommodate the limited vertical and horizontal space.

A 48‐year‐old male patient presented with a complaint of mobile and lingually displaced lower right mandibular central incisor, causing discomfort during mastication. There was no contributory medical or social history. Dental history revealed episodes of recurrent infection with a history of pus exudate from that tooth. Upon clinical examination, the mandibular right central incisor was found to be lingually positioned, supra‐erupted, with grade III mobility. Periodontal pocket depths ranged from 5 to 9 mm, and gingival recession of 3–5 mm on six points pocket charting. The adjacent teeth were checked for pocketing and mobility with no significant pathology. Radiographic examination revealed the loss of alveolar bone support beyond the apical region (Figure 2A). The diagnosis for tooth 41 was localized periodontitis, and with the loss of bone support up to the root length, the prognosis of the tooth was poor, and the treatment option for tooth 41 was for extraction. The mobile tooth was removed atraumatically, as shown in Figure 2B. Bonding of the acrylic tooth splint was carried out, and the patient expressed satisfaction with the aesthetics of the tooth splint. (Figure 2C,D).

### 1.3 Case 3

**FIGURE 3:**
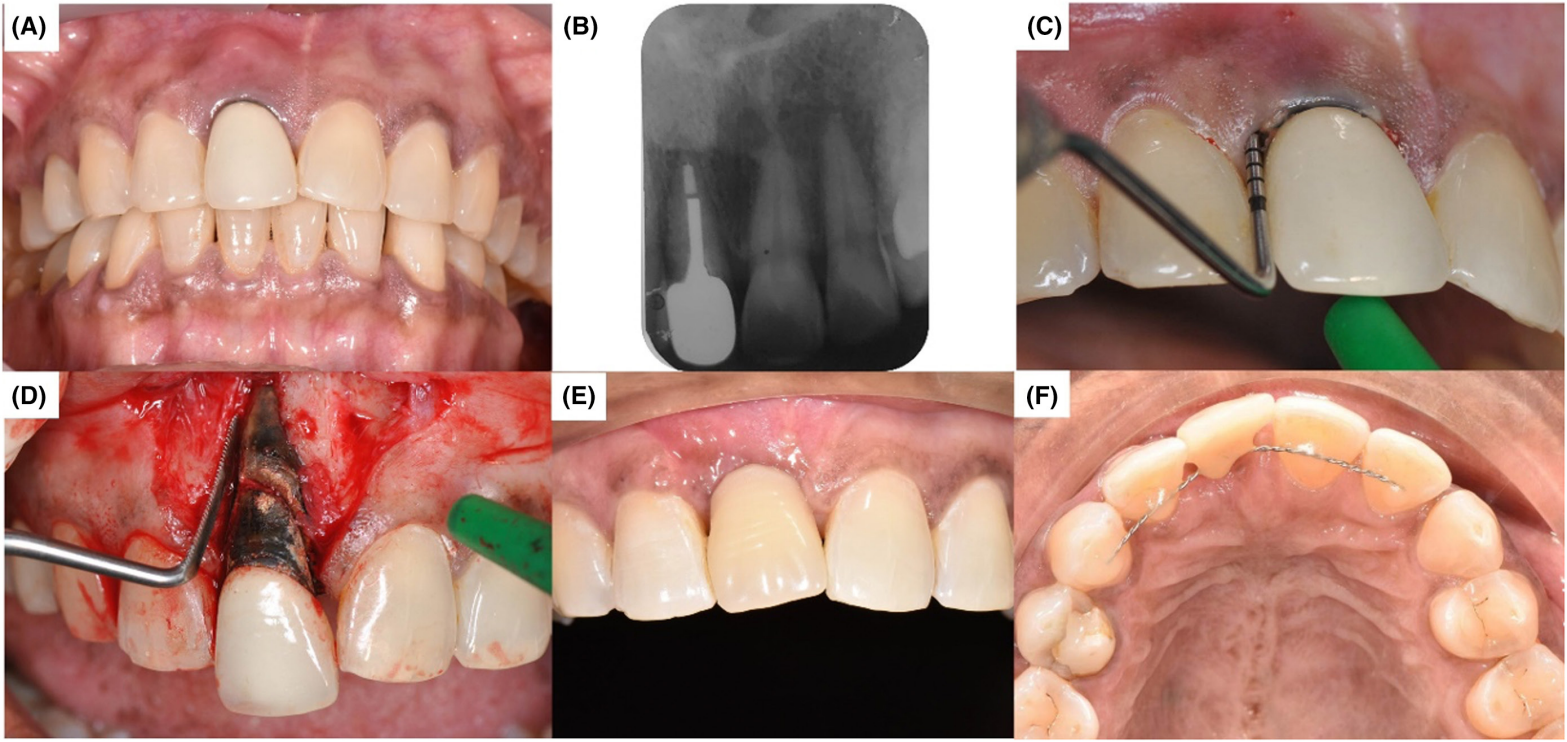
Clinical photographs from case 3. (A) Frontal view showing tooth 21 before extraction with associated pus discharge and swollen labial mucosa. (B) Periapical radiograph of tooth 11 taken during the pre‐operative stage. (C) Localized 11 mm periodontal pocketing at distolabial of tooth 11. (D) Diagnosis of horizontal root fracture tooth 11 confirmed. (E) Frontal view showing the splinted acrylic tooth of 11 with acceptable aesthetics. (F) Upper occlusal view showing splinted acrylic tooth 11.

A 52‐year‐old female patient complained of a mobile crown on her upper right central incisor. The patient is fit and healthy. Dental history revealed multiple episodes of crown dislodgement and recementation dating back 7 years ago. Clinical examination of the affected tooth revealed grade III mobility, the presence of pus discharge, swollen labial mucosa and associated with tenderness upon palpation and percussion (Figure 3A). The periapical radiograph showed periapical radiolucency on tooth 11 and the gap between the root fill and post in the canal (Figure 3B). Periodontal probing revealed a localized pocketing of 11.0 mm at the disto‐labial region (Figure 3C). The tooth was diagnosed as previously endodontically treated with chronic periapical abscess of 11 secondary due to a fractured root. The diagnosis was confirmed when a flap was raised, and the fracture line was visibly noted (Figure 3D). The patient agreed to a ridge preservation procedure followed by implant placement to replace the missing 11 as the definitive treatment plan. The patient was prescribed an Essix retainer with acrylic tooth replacement immediately after surgery. However, the patient disliked the Essix retainer as she could not chew well on her other teeth and requested an alternative. Hence, the immediate bonding of an acrylic tooth splint was proposed and delivered to her. (Figure 3D,E).

### 1.4 Steps involved in the acrylic tooth splint technique

This technique requires a laboratory preparation stage before the tooth extraction to reduce the chairside time during the extraction appointment.

### 1.5 Laboratory stage

**FIGURE 4:**
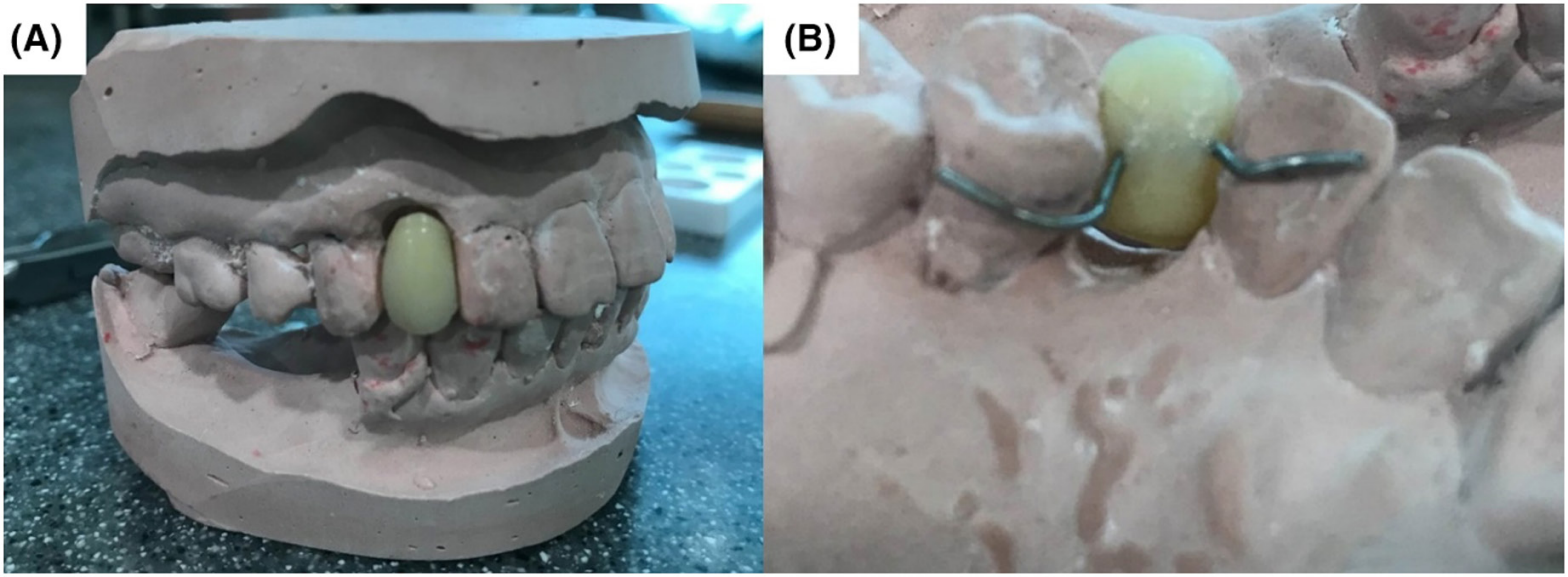
Laboratory steps. (A) Casted stone model showing single acrylic tooth splint in place (tooth 13). (B) Palatal view of the secured acrylic tooth using composite resin.

Step 1 A study model of the arch is used, and the impending tooth to be extracted is trimmed off to simulate post‐extraction socket contour. A replacement acrylic denture tooth is trimmed to fit into the horizontal and vertical space of the prepared edentulous area (Figure 4A).
Step 2 A slot/groove was prepared on the palatal/lingual surface of the acrylic tooth with an undercut to accommodate the diameter of 0.7 mm wire. The wire was bent to follow the curvature on the palatal surfaces of adjacent teeth, incorporating the acrylic tooth on the prepared wire.
Step 3 The bent wire was placed into the undercut slot prepared on the acrylic tooth and secured with composite resin and a bonding agent (Figure 4B).
Step 4 Occlusion was checked using articulating paper on static and dynamic movements to ensure no occlusal interference or any premature contacts that may contribute to the acrylic tooth splint dislodgement.

### 1.6 Clinical stage

After the extraction of the teeth and hemostasis is achieved, the acrylic tooth splint is adapted onto the lingual/palatal surfaces of the adjacent teeth and fixated onto adjacent teeth, which were treated using etch and rinse adhesive bonding and composite resin.

## 2 RESULTS

For all the cases discussed above, the patients reported a pleasing and satisfactory outcome related to the overall duration of the splint in the mouth. None of the patients experienced any dislodgements throughout the splint being in situ; for Case 1 (5 months), Case 2 (6 months), and Case 3 (5 months). All patients have received a definitive implant prosthesis and are currently in the maintenance phase of the treatment.

## 3 DISCUSSION

Immediate replacement of extracted anterior teeth can assuage the psychological and social impact of losing one's teeth.^15^ While numerous techniques are available for the immediate replacement of extracted teeth, each option should be evaluated for suitability in the clinical situation. Table 1 below outlines the advantages and disadvantages of these options.

**TABLE 1:** Advantages and disadvantages of extracted anterior tooth replacement option.

Options	Advantages	Disadvantages
Immediate denture replacing tooth^13^ ^,^ ^14^	Easy procedure Suitable in situations if no implants are planned at this site Occlusion is not affected much and the patient can chew on other teeth with ease Oral hygiene may be slightly affected as the denture would act as a food trap	Laboratory time and costs incurred If alveolar ridge preservation was done during the extraction appointment, the immediate denture may not fit very well and require further adjustments during that appointment Pressure from the dentures onto the healing mucosa during mastication can initiate the bone resorption process/loss of bone graft Speech impediment during the adaptive phase of wearing a new denture
2 Essix retainer with pontic tooth replacement ^16^ ^,^ ^17^	Easy to fabricate, possibly done in the clinic if the machine is available Because it is resting on occlusal surfaces of other teeth, it does not impinge on a healing extraction socket that may have been grafted during the extraction of the tooth	Difficult to eat properly with the retainer on. If a patient does eat with the retainer on, the retainer tends to perforate or crack easily due to plastic fatigue Cumbersome for patients to keep removing it and replacing it for meals Minor speech impediment during the initial adaptive phase of wearing a retainer
3 Use of extracted tooth as pontic, splinted to adjacent teeth using fiber^18^ ^,^ ^19^	High acceptance by patients as the appearance does not change much Fixed; patients need not remove them as in Options 1 and 2 Splinting may be beneficial for periodontally involved adjacent teeth	Additional chairside time is needed to section off the root from the crown and shaping it properly The extracted tooth may develop some malodour over time, especially if the pulp chamber canal is not sealed properly Fiber material can be slightly costly Needs to be removed and reattached again each time a procedure is required (e.g. implant placement, implant impression, etc)
4 Use of extracted tooth as pontic, splinted to adjacent teeth by wire^20^	Similar to option no. 3	Similar to option no. 3 except wires are likely to be cheaper
5 Fiber‐reinforced composite bridges^21^, ^22^, ^23^, ^24^	Similar to Option no. 3	Time‐consuming Costly (due to fibers and a large amount of composite needed) Needs to be removed and reattached again each time a procedure is required (e.g., implant placement, implant impression, etc)
6 Use of acrylic denture tooth splinted to adjacent teeth using fiber^25^	Acceptable appearance Fixed; patient need not remove them as in options 1 and 2 Shortened chairside time as compared to Options 3 and 4. Less likely to be malodorous as compared to Options 3 and 4 Splinting may be beneficial for periodontally involved adjacent teeth	Minimal laboratory time required‐ Fiber material can be slightly costly Needs to be removed and reattached again each time a procedure is required (e.g., implant placement, implant impression, etc)
7 Use of acrylic denture tooth splinted to adjacent teeth by wires^12^	Same as Option no. 5 Cost of wires cheaper than Option 5 (fibers)	Minimal laboratory time required Needs to be removed and reattached again each time a procedure is required (e.g. implant placement, implant impression, etc)

This case series highlighted the usage of acrylic tooth splinted with 0.6 mm, twisted, as reported in case 3‐ and 0.7‐mm diameter wires, untwisted, as seen in Cases 1 and 2. They were placed with composite on the palatal/lingual surface for provisionalisation of a missing/extracted anterior tooth. The advantages and disadvantages of this technique are summarized in Table 1, Option 7. Moreover, the composite resin's bond strength ensures the splint's longevity for as long as needed.^26^

In preparing the acrylic tooth to be used in the splint, it should ideally be contoured for an ovate pontic configuration on the tooth intaglio surface facing the gingiva to allow the gingiva to heal around the ovate pontic, simulating the emergence profile of a natural tooth.^27^ The wire is then secured with composite on the palatal/lingual surfaces while the interproximal area between the pontic tooth and the natural tooth is bonded together using composite. To keep the gingiva healthy, sufficient embrasure space ensures the patient can clean the interproximal area underneath the splint. The acrylic tooth must be removed from occlusion to prevent dislodgement during the provisionalisation phase.

However, this technique of choice requires careful selection and consideration, namely, sufficient occlusal space on the palatal surfaces of the adjacent teeth to avoid interference in occlusion. It would not be suitable in cases with a deep overbite.^28^ It is unsuitable in crowded or mal‐aligned adjacent teeth, which may complicate splinting. For the canine tooth replacement, occlusion should preferably be in group function during the lateral excursion. Placement of the wire on the palatal/lingual surface must be at the interproximal contact point with the adjacent teeth if possible. Patients must be educated on proper oral hygiene care to prevent plaque accumulation due to splint placement. The patient must be warned on gentle usage of the involved area to avoid dislodgement. Patients with parafunctional habits may not be suitable for this technique.

## 4 CONCLUSION

The acrylic tooth splint described in this case series illustrates a simple yet reliable technique for replacing a single tooth in the aesthetic zone. With proper case selection to match the patient's requests and needs, this technique may be helpful for clinicians considering immediate replacement of single anterior teeth.

## AUTHOR CONTRIBUTIONS

**Fara Azwin Adam:** Conceptualization; data curation; investigation; methodology; writing – original draft. **Farah Syazwani Mohamd Tarmizi:** Conceptualization; data curation; formal analysis; investigation; methodology; writing – review and editing. **Chui Ling Goo:** Conceptualization; methodology; resources; supervision; validation; visualization; writing – review and editing.

## FUNDING INFORMATION

The manuscript was prepared with the support of the Incentive Grant for FGG Researchers, The National University of Malaysia (DD2020‐008).

## CONFLICT OF INTEREST STATEMENT

No known conflicts of interest are associated with this publication, and there has been no significant financial support for this work that could have influenced its outcome.

## CONSENT

All patients' written informed consent with permission to publish was obtained before publishing the article.

## Data Availability

Data sharing is not applicable to this article as no new data were created or analyzed in this study.
